# Editorial: New Advances in Electrocochleography for Clinical and Basic Investigation

**DOI:** 10.3389/fnins.2018.00310

**Published:** 2018-05-08

**Authors:** Martin Pienkowski, Oliver F. Adunka, Jeffery T. Lichtenhan

**Affiliations:** ^1^Salus University, Elkins Park, PA, United States; ^2^Wexner Medical Center, The Ohio State University, Columbus, OH, United States; ^3^School of Medicine, Washington University in St. Louis, St. Louis, MO, United States

**Keywords:** electrocochleography (ECochG), cochlea, hearing disorders, balance disorders, cochlear implants

Electrocochleography (ECochG) is a technique for recording evoked potentials from the inner ear, generally believed to originate from hair cells and nerve fibers. It is useful for assessing inner ear function in both laboratory and clinical settings. The abbreviation ECochG is preferable to ECoG, because the latter can be confused with “electrocorticogram” (Ferraro, 1986). ECochG measurements are typically made from the ear canal or eardrum (extratympanic), from the promontory or round window niche (transtympanic), or from inside the cochlea (intracochlear). Extratympanic ECochG recordings are most commonly made with “tiptrodes” (gold foil wrapped around insert earphones) or “tymptrodes” (electrodes placed directly on the tympanic membrane). While the amplitude of tymptrode measurements can be up to an order of magnitude larger than tiptrode measurements (Ferraro and Ferguson, 1989), transtympanic amplitudes can be far more than an order of magnitude larger than those on the eardrum (e.g., Ruth et al., 1988). We thus suggest that extratympanic measurements are best classified as far-field, and transtympanic measurements as near-field.

We will give a brief overview of ECochG before reviewing its traditional uses, and surveying recent advances that promise new applications in the assessment of auditory and vestibular function. References to the 23 papers collected for this Research Topic have been hyperlinked to Frontiers webpages. A more extensive historical overview of ECochG, including its basic features and applications, was provided by Eggermont. A complementary review by Gibson offers tips for optimizing ECochG recordings in different clinical situations. Electrovestibulography (EVestG) is an analogous emerging technique for characterizing vestibular hair cell and nerve function, and was reviewed by Brown et al.

Sensory cells of the inner ear can be manipulated, damaged, or destroyed in varying degrees depending on the ototoxic agent, administration approach, and dose, giving rise to hearing deficits at specific sound frequencies and intensities, as well as vestibular problems. A major long-term goal of ECochG is to help differentiate outer hair cell (OHC) from inner hair cell (IHC) or presynaptic losses, and from auditory nerve fiber (ANF) or postsynaptic losses, which are all presently lumped together as sensorineural hearing loss. Differential diagnosis of different forms of sensorineural hearing loss could prove useful in improving hearing aid fitting, in predicting cochlear implantation outcomes, and in individualized regenerative medicine (McLean et al., 2016, 2017).

ECochG measurements are believed to originate, in general, from at least four distinct cellular sources, the receptor potentials of OHCs and IHCs, and the dendritic potentials and spikes of ANFs. The phases or polarities of these components can vary along the cochlea in a complex fashion that depends on stimulus characteristics and electrode placement, confounding their separation and interpretation (Chertoff et al., 2012). For example, the origins of the commonly measured summating potential (SP) and cochlear microphonic (CM) are still unknown for the wide range of stimulus parameters and recording locations. The older term “cochlear response”, which seems to have become passé, thus remains an adequate descriptor of ECochG recordings as long as their origins remain elusive. A newer term with the same purpose appears to be the “total response” (e.g., McClellan et al., 2014). Continuing the progress toward untangling the different origins of ECochG measurements is essential to advance the clinical utility of ECochG (e.g., Forgues et al., 2014; Lichtenhan et al., 2014; Fontenot et al.).

The first ECochG measurements were obtained somewhat serendipitously by Wever and Bray (1930), who were attempting to record from cat ANFs. Their alternating or AC potential would come to be known as the cochlear microphonic (CM) and its origin was attributed to the hair cells, primarily to the more numerous and sensitive OHCs (Dallos and Cheatham, 1976), which amplify and sharpen sound-induced vibrations before their detection by the sensory IHCs and ANFs. It was later discovered that ANF spiking could also contribute to CM measurements, particularly in response to lower-frequency sounds (<1–2 kHz), and that IHCs contributed as well (Eggermont, 1974; Chertoff et al., 2002; Lichtenhan et al., 2014). This blend of responses became known as the auditory nerve neurophonic (ANN, e.g., Snyder and Schreiner, 1984; Forgues et al., 2014), which is simply a cochlear response to intense, low-frequency sounds. The Auditory Nerve Overlapped Waveform (ANOW; Lichtenhan et al., 2013, 2014) differs from the ANN in that it is evoked by low to moderate level sounds, and its cellular and spatial origins are known. ECochG measurements can be DC-biased by the summating potential (SP), and show compound action potential (CAP) responses to stimulus onsets and sometimes offsets, reflecting the synchronous spiking of ANFs (Davis et al., 1958; Ruben et al., 1961). The CAP is wave I of the auditory brainstem response (ABR), first characterized by Jewett and Williston (1971).

A long-standing use of ECochG has been to objectively corroborate a symptomatic and case-history diagnosis of endolymphatic hydrops in Meniere's disease and other pathological states (endolymphatic hydrops is not limited to Meniere's). In ears with endolymphatic hydrops, the SP/CAP ratio can be increased, due mainly to an increase in the SP, but also to a decrease in the CAP. Despite much research, it is not known whether the sensitivity and specificity of ECochG for detecting endolymphatic hydrops is high enough to be useful for individual patients. Sass (1998) reported high sensitivity and specificity (87 and 100%, respectively) when transtympanic click and 1 kHz tone burst SP/CAP ratios were combined with the increased CAP latency difference between rarefaction and condensation stimulus clicks that is also typical of ears with endolymphatic hydrops. Others have also reported good sensitivity and specificity by using the SP/CAP area (e.g., Ferraro, 2010). As reviewed by Eggermont and Hornibrook, the results of some other studies have been less encouraging, but there is consensus that tone burst ECochG presently yields the best results (Hornibrook). In a promising new approach, Lichtenhan et al.induced endolymphatic hydrops in guinea pigs using three classical manipulations and found that changes in the ANOW were more sensitive to small degrees of endolymphatic hydrops than were changes in traditional measures such as CAP thresholds and the endocochlear potential, suggesting that the ANOW could be useful in the early detection of endolymphatic hydrops.

ECochG can be used in the diagnosis of auditory neuropathy (Widen et al., 1995; Rance and Starr, 2015), an umbrella term that includes many etiologies such as drug- or hypoxia-induced IHC loss (Harrison, 1998; Salvi et al.), noise- and age-related synaptopathy (Kujawa and Liberman, 2015), hereditary synaptopathy and neuropathy (e.g., mutations of *OTOF, OPA1*, and other genes; Santarelli et al., 2013), and even acoustic neuroma. While MRI can be useful in confirming some cases of auditory neuropathy (e.g., Roche et al., 2010), it is typically diagnosed when an absent or abnormal CAP or ABR, even at high stimulus levels, co-occurs with a robust CM and/or otoacoustic emissions (OAEs). Speech perception deficits, both in quiet and in noise, are worse than expected from the audiometric loss. Identifying ears with auditory neuropathy is important for predicting cochlear implant outcomes, which are generally poorer compared to non-neuropathic patients (McMahon et al., 2008; Walton et al., 2008; Harrison et al., 2015; Santarelli et al., 2015).

Salvi et al.provided an instructive review of selective IHC loss in chinchillas due to the cancer drug carboplatin. Substantial IHC loss had no measurable effect on OAEs or the CM (however, see Chertoff et al., 2002), but reduced SP and CAP amplitudes. Tone thresholds in quiet were unaffected by IHC losses of up to 80%, but thresholds in noise were elevated (Lobarinas et al., 2016). Importantly, the chinchilla carboplatin studies reviewed by Salvi et al. were also among the first to provide compelling evidence for synaptic gain increases in the central auditory system in response to decreased peripheral input. While increased central gain can lead to improved audibility in quiet conditions (see e.g.,Hoben et al.), it might also lead to potentially bothersome tinnitus and hyperacusis (Noreña, 2011; Schaette and McAlpine, 2011; Pienkowski et al., 2014; Brotherton et al., 2015; Paul et al., 2017).

ECochG is a promising candidate for detecting noise- and age-related cochlear synaptopathy (Kujawa and Liberman, 2009, 2015; Sergeyenko et al., 2013). It was recently reported that college student musicians with normal audiometric thresholds up to 8 kHz, but mild hearing losses at 10–16 kHz, showed significantly increased click-evoked SP amplitudes and slightly decreased CAP amplitudes (Liberman et al., 2016), changes reminiscent of endolymphatic hydrops but in this case attributed to noise-induced synaptopathy. Bramhall et al. (2017) found reduced CAP amplitudes in military veterans with high noise exposure histories, and in non-veterans who reported a history of firearm use, compared with veterans and non-veterans with lower noise histories. Importantly, the reduced CAP amplitudes could not be explained by OHC dysfunction, as assessed with distortion product OAEs (DPOAEs). Other studies using CAP or ABR wave I amplitudes (as well as other metrics) have failed to detect evidence of synaptopathy in noise-exposed adults (e.g., Prendergast et al., 2017). However, it may be that people who regularly subject themselves to high recreational noise doses do so because of their “tougher” ears, which sustain less damage than the potentially more “tender” ears of people who avoid loud music and noise (see e.g., Henderson et al., 1993 for a general discussion of this issue).

Grinn et al. reported CAP and DPOAE amplitudes, and Words-in-Noise (WIN) performance in a group of young adults before, and 1 and 7 days after a loud recreational event, typically a concert (average dose of 93 dB A for 4 h, range 73–104 dB A for 1.5–16 h). Consistent with the notion of tough vs. tender ears, there was no correlation between the noise dose and the amount of temporary threshold shift (TTS) measured across study participants. Most showed a 1 day TTS of <10 dB (with full recovery at 7 days), accompanied by correspondingly small but significant temporary decreases in WIN scores. DPOAE amplitudes were affected at 1 day but only at 6 kHz, whereas CAP amplitudes to clicks and 2–4 kHz tone bursts were not affected. These results argue against the development of synaptopathy after a single recreational noise dose, consistent with laboratory noise exposure that caused a TTS in humans (Lichtenhan and Chertoff, 2008). It is likely that a number of such exposures is needed to produce permanent damage in primates (Pienkowski, 2017; Valero et al., 2017).

To reduce the prevalence of noise-induced hearing loss, tinnitus, and hyperacusis, it would be helpful to identify those with especially tender ears. Maison and Liberman (2000) showed that the strength of the medial olivocochlear (MOC) efferent reflex in guinea pigs, as measured by the contralateral suppression of DPOAEs, was strongly correlated with lower TTS after acoustic trauma. Unfortunately, this finding has yet to be replicated in humans (e.g., Hannah et al., 2014). Smith et al.made measurements of chirp-evoked human CAPs, confirming the original finding that chirps yield larger CAP amplitudes than clicks (Chertoff et al., 2010). Smith et al. found that CAP amplitudes were more strongly contralaterally suppressible than were DPOAE amplitudes, similar to the results of previous animal and human studies (Puria et al., 1996; Lichtenhan et al., 2016). Verschooten et al. made progress in studying the human MOC reflex triggered by *ipsilateral* sound, by proposing how to separate MOC effects from the confounds of mechanical and neural masking.

This Research Topic reports innovations in recording techniques and signal processing that point to new potentially useful roles for ECochG in clinical practice (Charaziak et al.; Cook et al.; Kennedy et al.). Other innovations have noteworthy applications associated with cochlear implantation. Bester et al., Dalbert et al., Koka et al., and O'Connell et al., used ECochG to objectively assess residual, low-frequency acoustic hearing in ears implanted with hybrid electric-acoustic stimulation devices. He et al. comprehensively reviewed the electrically-evoked CAP or eCAP, including its applications in establishing implant candidacy, in intraoperative monitoring for electrode guidance, and in post-operative device programming and outcome assessment. Riggs et al. made intraoperative measurements from child and adult implantees with and without diagnosed auditory neuropathy, and found results consistent with better hair cell but poorer neural function compared to non-neuropathic patients. While it remains a challenge to accurately estimate ANF survival in implant candidates, Pardo-Jadue et al. suggest that tymptrode measurements of spontaneous ANF firing (in the absence of sound or other stimulation) could be helpful in this regard.

The telemetric innovations of modern cochlear implants have advanced research in intracochlear ECochG. Kim et al. reported the first intracochlear ECochG measurements from cochlear implant (Nucleus Hybrid L24) users. Koka and Litvak performed the first intracochlear ECochG recordings in response to simultaneous electrical and acoustic stimulation in patients implanted with Advanced Bionics HiRes 90K Advantage. The results of these pioneering measurements may point the way forward to objectively programming hybrid cochlear implants and better predicting speech outcomes.

The past informs the present, as the saying goes, and this is certainly true of the field of ECochG. It is usual for even good data to be misinterpreted in the context of the available theories of the day. Likewise, it is usual for previous interpretations to become outdated as new advances are made. Nevertheless, interpretations, not data, are typically the main intellectual drive of textbooks and review articles, and new trainees to a field often begin with these sources. Once a knowledge base becomes firmly entrenched, it can sometimes be difficult and uncomfortable to realize that a framework is no longer adequate to encapsulate new findings, and needs updating. We hope to have clarified some of the main ideas, terminology, and origins of ECochG measurements, and encourage all to study the almost 90 year history of this field.

## Author contributions

MP: drafted the manuscript; JL and MP: edited the manuscript; JL: organized the Research Topic; JL, OA, and MP: shared editing responsibilities on the Research Topic.

### Conflict of interest statement

The authors declare that the research was conducted in the absence of any commercial or financial relationships that could be construed as a potential conflict of interest.

## References

[B1] Bramhall N. F. Konrad-Martin D. McMillan G. P. Griest S. E. (2017). Auditory brainstem response altered in humans with noise exposure despite normal outer hair cell function. Ear Hear. 38, e1–e12. 10.1097/AUD.0000000000000370 27992391 PMC5313078

[B2] Brotherton H. Plack C. J. Maslin M. Schaette R. Munro K. J. (2015). Pump up the volume: could excessive neural gain explain tinnitus and hyperacusis? Audiol. Neurootol. 20 273–282. 10.1159/000430459 26139435

[B3] Chertoff M. E. Amani-Taleshi D. Guo Y. Burkard R. (2002). The influence of inner hair cell loss on the instantaneous frequency of the cochlear microphonic. Hear. Res. 174, 93–100. 12433400 10.1016/s0378-5955(02)00642-1

[B4] Chertoff M. E. Earl B. R. Diaz F. J. Sorensen J. L. (2012). Analysis of the cochlear microphonic to a low-frequency tone embedded in filtered noise. J. Acoust. Soc. Am. 132, 3351–3362. 10.1121/1.4757746 23145616 PMC3505208

[B5] Chertoff M. E. Lichtenhan J. Willis M. (2010). Click- and chirp-evoked human compound action potentials. J Acoust Soc Am. 127, 2992–2996. 10.1121/1.2967890 21117748 PMC3188627

[B6] Dallos P. Cheatham M. A. (1976). Production of cochlear potentials by inner and outer hair cells. J. Acoust. Soc. Am. 60, 510–512. 10.1121/1.381086 993471

[B7] Davis H. Deatherage B. H. Eldredge D. H. Smith C. A. (1958). Summating potentials of the cochlea. Am. J. Physiol. 195, 251–261. 13583157 10.1152/ajplegacy.1958.195.2.251

[B8] Eggermont J. J. (1974). Basic principles for electrocochleography. Acta. Otolaryngol. Suppl. 316, 7–16. 4525558 10.1080/16512251.1974.11675742

[B9] Ferraro J. A. (1986). Electrocochleography. Semin. Hear. 7, 239–240.

[B10] Ferraro J. A. (2010). Electrocochleography: a review of recording approaches, clinical applications, and new findings in adults and children. J. Am. Acad. Audiol. 21, 145–152. 10.3766/jaaa.21.3.2 20211118

[B11] Ferraro J. A. Ferguson R. (1989). Tympanic ECochG and conventional ABR: a combined approach for the identification of wave I and the I-V interwave interval. Ear. Hear. 10, 161–166. 2744251

[B12] Forgues M. Koehn H. A. Dunnon A. K. Pulver S. H. Buchman C. A. Adunka O. F.. (2014). Distinguishing hair cell from neural potentials recorded at the round window. J. Neurophysiol. 111, 580–593. 10.1152/jn.00446.2013 24133227 PMC3921406

[B13] Hannah K. Ingeborg D. Leen M. Annelies B. Birgit P. Freya S.. (2014). Evaluation of the olivocochlear efferent reflex strength in the susceptibility to temporary hearing deterioration after music exposure in young adults. Noise Health 16, 108–115. 10.4103/1463-1741.132094 24804715

[B14] Harrison R. V. (1998). An animal model of auditory neuropathy. Ear. Hear. 19, 355–361. 9796644 10.1097/00003446-199810000-00002

[B15] Harrison R. V. Gordon K. A. Papsin B. C. Negandhi J. James A. L. (2015). Auditory neuropathy spectrum disorder (ANSD) and cochlear implantation. Int. J. Pediatr. Otorhinolaryngol. 79, 1980–1987. 10.1016/j.ijporl.2015.10.006 26545793

[B16] Henderson D. Subramaniam M. Boettcher F. A. (1993). Individual susceptibility to noise-induced hearing loss: an old topic revisited. Ear. Hear. 14, 152–168. 8344472 10.1097/00003446-199306000-00002

[B17] Jewett D. L. Williston J. S. (1971). Auditory-evoked far fields averaged from the scalp of humans. Brain 94, 681–696. 5132966 10.1093/brain/94.4.681

[B18] Kujawa S. G. Liberman M. C. (2009). Adding insult to injury: cochlear nerve degeneration after “temporary” noise-induced hearing loss. J. Neurosci. 29, 14077–14085. 10.1523/JNEUROSCI.2845-09.2009 19906956 PMC2812055

[B19] Kujawa S. G. Liberman M. C. (2015). Synaptopathy in the noise-exposed and aging cochlea: primary neural degeneration in acquired sensorineural hearing loss. Hear. Res. 330, 191–199. 10.1016/j.heares.2015.02.009 25769437 PMC4567542

[B20] Liberman M. C. Epstein M. J. Cleveland S. S. Wang H. Maison S. F. (2016). Toward a differential diagnosis of hidden hearing loss in humans. PLoS ONE 11:e0162726. 10.1371/journal.pone.0162726 27618300 PMC5019483

[B21] Lichtenhan J. T. Chertoff M. E. (2008). Temporary hearing loss influences post-stimulus time histogram and single neuron action potential estimates from human compound action potentials. J. Acoust. Soc. Am. 123, 2200–2212. 10.1121/1.2885748 18397026 PMC2811543

[B22] Lichtenhan J. T. Cooper N. P. Guinan J. J. Jr. (2013). A new auditory threshold estimation technique for low frequencies: proof of concept. Ear. Hear. 34, 42–51. 10.1097/AUD.0b013e31825f9bd3 22874644 PMC3495092

[B23] Lichtenhan J. T. Hartsock J. J. Gill R. M. Guinan J. J. Jr. Salt A. N. (2014). The auditory nerve overlapped waveform (ANOW) originates in the cochlear apex. J. Assoc. Res. Otolaryngol. 15, 395–411. 10.1007/s10162-014-0447-y 24515339 PMC4010591

[B24] Lichtenhan J. T. Wilson U. S. Hancock K. E. Guinan J. J. Jr. (2016). Medial olivocochlear efferent reflex inhibition of human cochlear nerve responses. Hear. Res. 333, 216–224. 10.3109/14992027.2015.1122238 26364824 PMC4788580

[B25] Lobarinas E. Salvi R. Ding D. (2016). Selective inner hair cell dysfunction in chinchillas impairs hearing-in-noise in the absence of outer hair cell loss. J. Assoc. Res. Otolaryngol. 17, 89–101. 10.1007/s10162-015-0550-8 26691159 PMC4791417

[B26] Maison S. F. Liberman M. C. (2000). Predicting vulnerability to acoustic injury with a noninvasive assay of olivocochlear reflex strength. J. Neurosci. 20, 4701–4707. 10.1523/JNEUROSCI.20-12-04701.2000 10844039 PMC6772446

[B27] McClellan J. H. Formeister E. J. Merwin W. H. III. Dillon M. T. Calloway N. Iseli C.. (2014). Round window electrocochleography and speech perception outcomes in adult cochlear implant subjects: comparison with audiometric and biographical information. Otol. Neurotol. 35, e245–e252. 10.1097/MAO.0000000000000557 25118584

[B28] McLean W. J. McLean D. T. Eatock R. A. Edge A. S. (2016). Distinct capacity for differentiation to inner ear cell types by progenitor cells of the cochlea and vestibular organs. Development 143, 4381–4393. 10.1242/dev.139840 27789624 PMC5201044

[B29] McLean W. J. Yin X. Lu L. Lenz D. R. McLean D. Langer R.. (2017). Clonal expansion of Lgr5-positive cells from mammalian cochlea and high-purity generation of sensory hair cells. Cell. Rep. 18, 1917–1929. 10.1016/j.celrep.2017.01.066 28228258 PMC5395286

[B30] McMahon C. M. Patuzzi R. B. Gibson W. P. Sanli H. (2008). Frequency-specific electrocochleography indicates that presynaptic and postsynaptic mechanisms of auditory neuropathy exist. Ear. Hear. 29, 314–325. 10.1097/AUD.0b013e3181662c2a 18344874

[B31] Noreña A. J. (2011). An integrative model of tinnitus based on a central gain controlling neural sensitivity. Neurosci. Biobehav. Rev. 35, 1089–1109. 10.1016/j.neubiorev.2010.11.003 21094182

[B32] Paul B. T. Bruce I. C. Roberts L. E. (2017). Evidence that hidden hearing loss underlies amplitude modulation encoding deficits in individuals with and without tinnitus. Hear. Res. 344, 170–182. 10.1016/j.heares.2016.11.010 27888040

[B33] Pienkowski M. (2017). On the etiology of listening difficulties in noise despite clinically normal audiograms. Ear. Hear. 38, 135–148. 10.1097/AUD.0000000000000388 28002080 PMC5325255

[B34] Pienkowski M. Tyler R. S. Roncancio E. R. Jun H. J. Brozoski T. Dauman N. (2014). A review of hyperacusis and future directions: part II. Measurement, mechanisms, and treatment. Am. J. Audiol. 23, 420–436. 10.1044/2014_AJA-13-0037 25478787

[B35] Prendergast G. Guest H. Munro K. J. Kluk K. Léger A. Hall D. A.. (2017). Effects of noise exposure on young adults with normal audiograms I: Electrophysiology. Hear. Res. 344, 68–81. 10.1016/j.heares.2016.10.028 27816499 PMC5256477

[B36] Puria S. Guinan J. J. Jr. Liberman M. C. (1996). Olivocochlear reflex assays: effects of contralateral sound on compound action potentials versus ear-canal distortion products. J. Acoust. Soc. Am. 99, 500–507. 8568037 10.1121/1.414508

[B37] Rance G. Starr A. (2015). Pathophysiological mechanisms and functional hearing consequences of auditory neuropathy. Brain 138, 3141–3158. 10.1093/brain/awv270 26463676

[B38] Roche J. P. Huang B. Y. Castillo M. Bassim M. K. Adunka O. F. Buchman C. A. (2010). Imaging characteristics of children with auditory neuropathy spectrum disorder. Otol. Neurotol. 31, 780–788. 20593543 10.1097/mao.0b013e3181d8d528 PMC3664382

[B39] Ruben R. J. Bordley J. E. Lieberman A. T. (1961). Cochlear potentials in man. Laryngoscope 71, 1141–1164. 10.1288/00005537-196110000-00001 14494859

[B40] Ruth R. A. Lambert P. R. Ferraro J. A. (1988). Electrocochleography: methods and clinical applications. Am. J. Otol. Suppl, 9, 1–11. 3059811

[B41] Santarelli R. del Castillo I. Starr A. (2013). Auditory neuropathies and electrocochleography. Hear. Bal. Commun. 11, 130–137. 10.3109/21695717.2013.815446

[B42] Santarelli R. Rossi R. Scimemi P. Cama E. Valentino M. L. LaMorgia C.. (2015). OPA1-related auditory neuropathy: site of lesion and outcome of cochlear implantation. Brain 138, 563–576. 10.1093/brain/awu378 25564500 PMC4339771

[B43] Sass K. (1998). Sensitivity and specificity of transtympanic electrocochleography in Ménière's disease. Acta Otolaryngol. 118, 150–156. 10.1080/00016489850154838 9583780

[B44] Schaette R. McAlpine D. (2011). Tinnitus with a normal audiogram: physiological evidence for hidden hearing loss and computational model. J. Neurosci. 31, 13452–13457. 10.1523/JNEUROSCI.2156-11.2011 21940438 PMC6623281

[B45] Sergeyenko Y. Lall K. Liberman M. C. Kujawa S. G. (2013). Age-related cochlear synaptopathy: an early-onset contributor to auditory functional decline. J. Neurosci. 33, 13686–13694. 10.1523/JNEUROSCI.1783-13.2013 23966690 PMC3755715

[B46] Snyder R. L. Schreiner C. E. (1984). The auditory neurophonic: basic properties. Hear. Res. 15, 261–280. 6501114 10.1016/0378-5955(84)90033-9

[B47] Valero M. D. Burton J. A. Hauser S. N. Hackett T. A. Ramachandran R. Liberman M. C. (2017). Noise-induced cochlear synaptopathy in rhesus monkeys (Macaca mulatta). Hear. Res. 353, 213–223. 10.1016/j.heares.2017.07.003 28712672 PMC5632522

[B48] Walton J. Gibson W. P. Sanli H. Prelog K. (2008). Predicting cochlear implant outcomes in children with auditory neuropathy. Otol. Neurotol. 29, 302–309. 10.1097/MAO.0b013e318164d0f6 18317399

[B49] Wever E. G. Bray C. W. (1930). Auditory nerve impulses. Science 71, 215. 10.1126/science.71.1834.215 17818230

[B50] Widen J. E. Ferraro J. A. Trouba S. E. (1995). Progressive neural hearing impairment: case report. J. Am. Acad. Audiol. 6, 217–224. 7620198

